# T7 phage-assisted evolution of riboswitches using error-prone replication and dual selection

**DOI:** 10.1038/s41598-024-52049-9

**Published:** 2024-01-29

**Authors:** Eduardo Goicoechea Serrano, Carlos Blázquez-Bondia, Alfonso Jaramillo

**Affiliations:** 1 Warwick Integrative Synthetic Biology Centre and School of Life Sciences, University of Warwick, Coventry, CV4 7AL UK; 2 London BioFoundry, Imperial College Translation & Innovation Hub, White City Campus, 84 Wood Lane, London, W12 0BZ UK; 3 De novo Synthetic Biology Lab, i2sysbio, CSIC-University of Valencia, Parc Científic Universitat de València, Calle Catedrático Agustín Escardino, 9, 46980 Paterna, Spain

**Keywords:** Synthetic biology, Prokaryote

## Abstract

Leveraging riboswitches, non-coding mRNA fragments pivotal to gene regulation, poses a challenge in effectively selecting and enriching these functional genetic sensors, which can toggle between ON and OFF states in response to their cognate inducers. Here, we show our engineered phage T7, enabling the evolution of a theophylline riboswitch. We have replaced T7’s DNA polymerase with a transcription factor controlled by a theophylline riboswitch and have created two types of host environments to propagate the engineered phage. Both types host an error-prone T7 DNA polymerase regulated by a T7 promoter along with another critical gene—either cmk or pifA, depending on the host type. The cmk gene is necessary for T7 replication and is used in the first host type for selection in the riboswitch's ON state. Conversely, the second host type incorporates the pifA gene, leading to abortive T7 infections and used for selection in the riboswitch’s OFF state. This dual-selection system, termed T7AE, was then applied to a library of 65,536 engineered T7 phages, each carrying randomized riboswitch variants. Through successive passage in both host types with and without theophylline, we observed an enrichment of phages encoding functional riboswitches that conferred a fitness advantage to the phage in both hosts. The T7AE technique thereby opens new pathways for the evolution and advancement of gene switches, including non-coding RNA-based switches, setting the stage for significant strides in synthetic biology.

## Introduction

Phages have transformed the directed evolution of genetic systems with their robust tolerance for high mutagenesis loads and swift replication rates. Phage Assisted Continuous Evolution (PACE) technology^1^, leveraging the M13 phage, fast-tracks protein evolution in continuous cultures. This method substitutes an essential gene in the M13 phage genome with an inactively transcribed version in the host, allowing any gene of interest (GOI) to evolve if it can trigger the transcription of the critical gene. The PACEmid^2^ technology, a subsequent refinement of the PACE approach, moves almost all phage genes to the host, stopping these genes' adaptation. PACEmid aids the evolution of satellite phages by packaging plasmids as functional virions, or phagemids, using the phage's packaging signal, proving particularly beneficial for GOIs with minimal initial activity. For example, it evolved the cro repressor into a transcription factor activator, creating the smallest known transcription factor activator of just 63 amino acids. PACEmid also increases the non-specific mutation rate tenfold. However, the use of M13 phage in bioreactors poses challenges. Post-infection, bacterial hosts survive, releasing non-evolved phages from biofilm-producing cells, a problem that intensifies under strict selection conditions. This becomes especially troublesome when the initial GOI activity is low or the GOI exhibits significant activity changes depending on the chemical environment, as is the case with gene switches.

We present T7AE, a new genetic selection system that utilizes phage T7 for the phage-assisted evolution of gene switches, notably riboswitches. Departing from prior reliance on M13 phage, we use the lytic T7 phage to spur the evolution of gene switches composed of non-coding RNA fragments. While earlier research primarily focused on evolving protein-based systems, non-coding RNA switches have seen significant development through computational design methods^3–6^. Our study addresses this disparity, expanding phage-assisted evolution techniques to include gene switches. This extension provides a powerful experimental platform for the directed evolution of gene switches. Through combining phage-assisted evolution, predominantly applied to proteins, with the distinctive attributes of the lytic phage T7, we seek to uncover the evolutionary potential of gene switches.

Riboswitches, specific regions within mRNA molecules, possess a unique tertiary structure that impacts downstream gene expression^7^. Binding to small molecules triggers a structural rearrangement in riboswitches, leading to modifications in the secondary structure of the succeeding mRNA sequence, either forming a new transcriptional terminator or revealing previously hidden ribosome binding sites (RBS)^8^. Although riboswitches frequently occur in archaea and prokaryotes, they've been noted in eukaryotes and even prophages, as well as engineered to operate in viruses^9,10^. Their cross-species functionality and significant mutability make riboswitches compelling research subjects, offering versatile applications and opportunities for generating novel variants that no longer recognize original ligands^11^. Additionally, riboswitches’ compact size and clear operational mechanism make them one of the simplest genetic switches, illuminating principles of gene regulation. Investigation into riboswitches’ characteristics and applications holds immense promise for advancing synthetic biology and genetic engineering. Researchers have strived to uncover new riboswitches^12^, enhance their functionality^13^, and even design them De novo^14^ due to their potential. Current methods for creating novel riboswitches include computer modeling^15^, directed evolution^16^, and De novo design^17^. While many approaches concentrate on In vitro selection, requiring subsequent In vivo validation, there are instances of In vivo methods^18^, which allow for the screening of viable riboswitch candidates early on, broadening the sequence space and amplifying the riboswitch’s final activity. The development of diverse and modular selection circuits is key to overcoming these challenges^19,20^.

In this study, we focus on the accelerated evolution of genetic switches using dual selection methods, a critical process for effectively selecting both active and inactive genes in environments that induce ON and OFF states^21^. Our primary objective is to engineer new riboswitches using lytic phages as the evolutionary platform. The theophylline riboswitch, our gene of interest, gets incorporated into the T7 phage genome and subsequently randomized using phage genome engineering techniques^22^. This work represents the first instance of a functional riboswitch integration into a phage genome, setting it apart from prior studies identifying ribozymes in phages^23^. We aim to evolve these riboswitches through T7AE and analyze the resulting phage population. The report is divided into four primary sections: an introduction to T7AE, detailing our dual selection method for directed evolution via T7 phage; a description of our riboswitch phage library; a presentation of our dual selection cycles; and finally, an estimation of riboswitch activity from the phage phenotype which demonstrates riboswitch adaptation during evolution. We follow these sections with a detailed discussion. In our experimental process, a varied phage population carrying randomized theophylline riboswitches undergoes several cycles of dual selection, leading to a noticeable reduction in riboswitch diversity within the phage population. This process also results in the emergence of new riboswitches with notable activation changes between selection stages. Our assessment of riboswitch sequence variability and measurement of phage activity under different inducer conditions offers insights into the evolutionary dynamics of riboswitches within the phage population and their functional responses to inducers.

## Results

### T7AE: dual selection method for directed evolution via T7 phage

**Figure 1:**
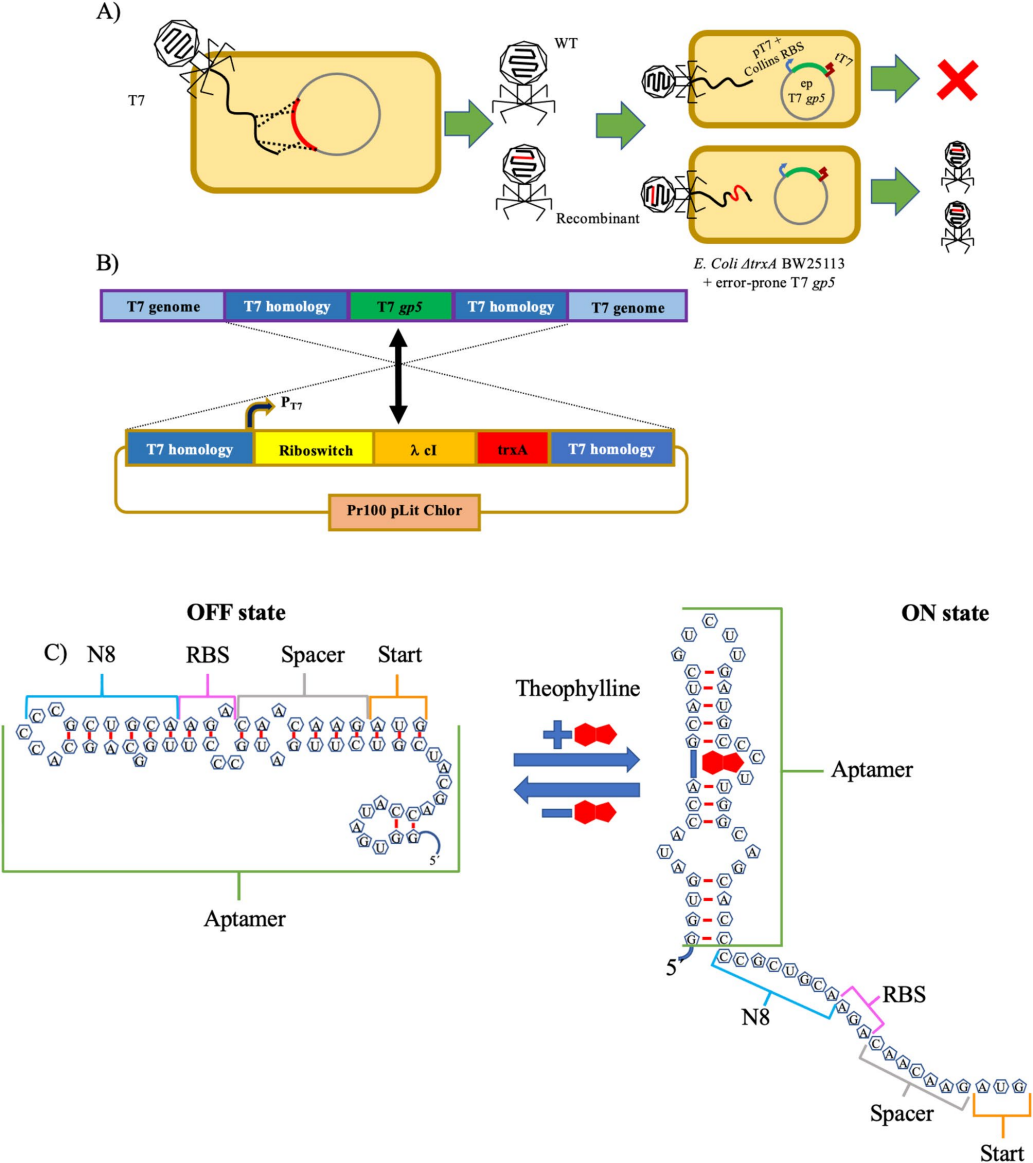
Engineering a riboswitch in the T7 phage using trxA marker. (**A**) Obtaining recombinant phages: A schematic representation of the homologous recombination process occurring within the phage population. Recombinant phages are generated as a result, and these recombinants are selected by infecting a specific replication strain. (**B**) Detail of genetic constructs: Depiction of the T7 genome and the recombination plasmid, highlighting the homology regions and the elements they flank. Homologous recombination enables the swapping of these two fragments. (**C**) Versions of the theophylline riboswitch: Illustration of the “OFF” state (left) and the “ON” state (right) of the theophylline riboswitch.

We engineer the T7 phage genome and its *Escherichia coli* host to establish an error-prone phage replication mechanism for implementing our T7AE system. We remove the DNA polymerase gene (gp5) on the T7 phage genome and replace it with a cI transcription factor from the λ phage, regulated by a theophylline riboswitch at its 5′ UTR. On the host side, we use an error-prone variant of gp5^24^ to enhance the T7 phage mutation rate. We also introduce a gene for the selection or counter-selection of functional or non-functional riboswitches, respectively. Our approach of combining an error-prone phage replication system with positive and negative selection steps is designed to encourage the emergence of novel, functionally enhanced riboswitch variants. This strategy, aimed at broadening our understanding of non-coding RNA engineering, allows for the experimental evolution of riboswitches. We outline our T7 phage-based screening strategy for novel riboswitch sequences in Fig. 1. Utilizing homologous recombination^22,25^ (Fig. 1A), we integrate our DNA fragment of interest into the T7 phage genome. We create plasmids containing phage genome-homologous regions, aiming to remove the phage's gp5 gene. The plasmids house either a positive control riboswitch sequence (PAJ341), or a library of riboswitch variants (PAJ342), as depicted in Fig. S4 (further details in Supplementary Materials). The recombined gene fragment, carrying multiple elements (Fig. 1B), includes a T7 promoter, a theophylline riboswitch version^26^ (Fig. 1C), the λ phage-derived cI transcription factor^27^, and the trxA gene, essential for phage replication. The trxA serves as a cloning marker to identify recombinant T7 genomes^22^. During selection, the λ cI repressor activates the PRM promoter^28^, which controls downstream gene expression. This configuration integrates the theophylline riboswitch-controlled gene expression system into the T7 phage genome, enabling the study and evolution of riboswitches within the framework of phage-assisted evolution.

**Figure 2:**
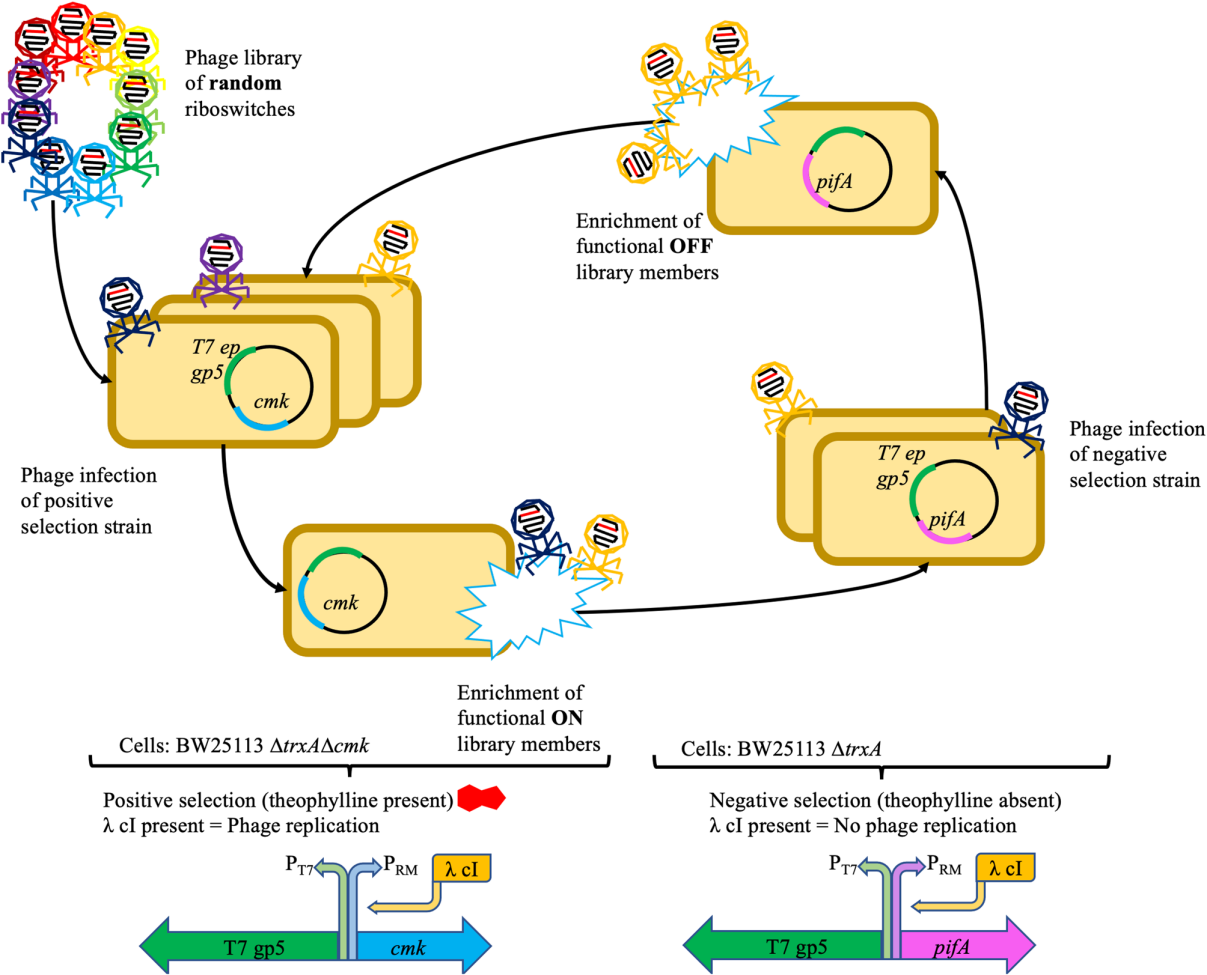
Schema showing the design and use of our T7AE system. General structure of the full phage evolutionary procedure: (1) phage library infection: a phage library comprising various riboswitch variants infects a positive selection strain containing a positive selection plasmid. (2) Positive selection step: in the presence of theophylline, only phages carrying functional variants of the riboswitch will be able to replicate effectively. (3) Negative selection step: the surviving phages from the positive selection are subjected to a negative selection step. In the absence of theophylline, the riboswitches should remain inactive. However, to eliminate constitutively active phages, the pifA gene is transcribed, rendering these phages unable to replicate effectively. (4) Cycling between positive and negative selections: the resulting phages from the negative selection step infect the positive selection strain once again, initiating another cycle. This iterative process progressively exerts evolutionary pressure, driving the selection and improvement of functional riboswitch variants over time. The T7AE system combines positive and negative selection steps to enforce evolutionary pressure, facilitating the development and enhancement of riboswitches within the phage population.

To facilitate the evolution of a conditional genetic device, we employed a dual selection strategy consisting of positive and negative stages^29,30^ (Fig. 2). For the positive selection phase, we added a high concentration of theophylline during phage replication, with the riboswitch controlling the replication rate via λ cI, thereby determining the selection outcome. We infected an engineered *Escherichia coli* strain, primed for positive selection, with our T7 phage carrying the riboswitch. This strain, carrying a cmk gene knockout essential for T7 phage replication^24^ but not *Escherichia coli* growth, was transformed with a plasmid encoding the PRM promoter regulating the cmk gene. Theophylline's presence selectively favored the phages carrying an active riboswitch, as these were the only ones capable of producing the λ cI necessary to activate the essential cmk gene.

In the negative selection phase, a different *Escherichia coli* strain, retaining the original cmk gene and transformed with a plasmid containing the PRM-regulated pifA gene, was used for infection. The pifA gene, originating from the F-plasmid, inhibits T7 replication^31^. This stage requires an absence of theophylline for the riboswitch to remain inactive. Any remaining riboswitch activity would escalate λ cI translation, thus activating the pifA gene and resulting in abortive infection. Consequently, phages carrying a riboswitch mutation that doesn't fully repress λ cI translation in theophylline's absence struggle to replicate properly. By alternating between positive and negative selection in theophylline's presence and absence respectively, we progressively screened the initial pool of randomly mutated riboswitches (65,536 or 4^8^ variants) to yield more efficient riboswitches. This strategy fosters the enrichment and selection of riboswitches with enhanced conditional behavior across selection cycles.

### Library design

**Figure 3:**
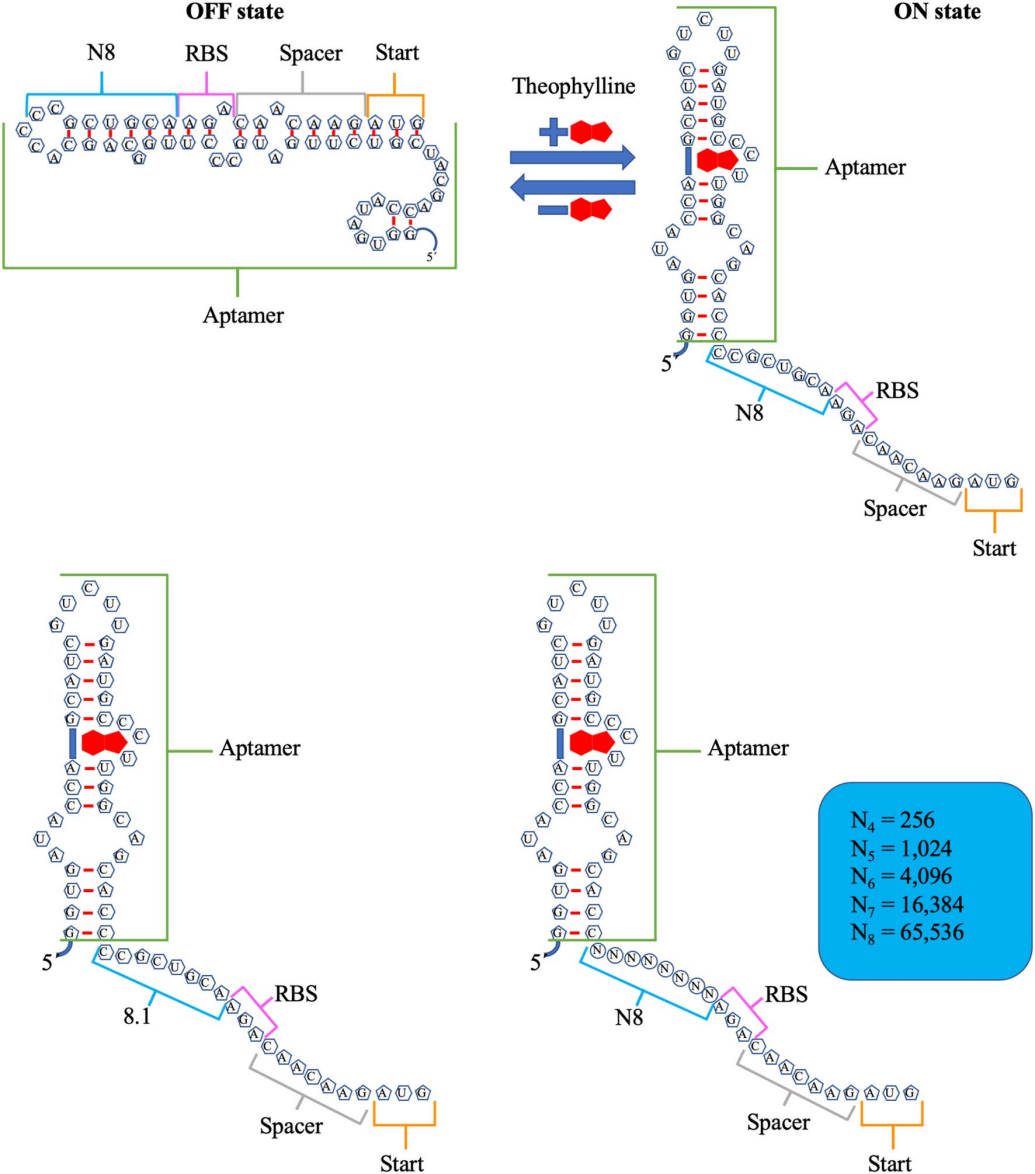
Design of riboswitch library. “OFF” state of the riboswitch (top left), “ON” state of the riboswitch (top right), 8.1 version of the riboswitch, which showed a 36-fold activation in a previous report^28^ (bottom left) and structure of the theoretical library obtained after randomizing the 8 nucleotides following the stem of the riboswitch (bottom right).

To foster a broad library of phages, we infused random mutations into an 8-nucleotide region of the riboswitch, chosen based on previous research^26^ (Fig. 3). The theoretical diversity of the library, considering all possible combinations of mutations in this 8-nucleotide region, encompasses 65,536 distinct variants. Given our transformation efficiency—measured at 1.11 * 10^6^ colony-forming units (cfu) per microgram (µg)—we accomplished a 17-fold coverage of the library with the utilized cultures (Fig. 3). This coverage implies a representation of the majority of library variants, thereby boosting the probability of unearthing novel and enhanced riboswitches through the evolutionary process.

**Figure 4:**
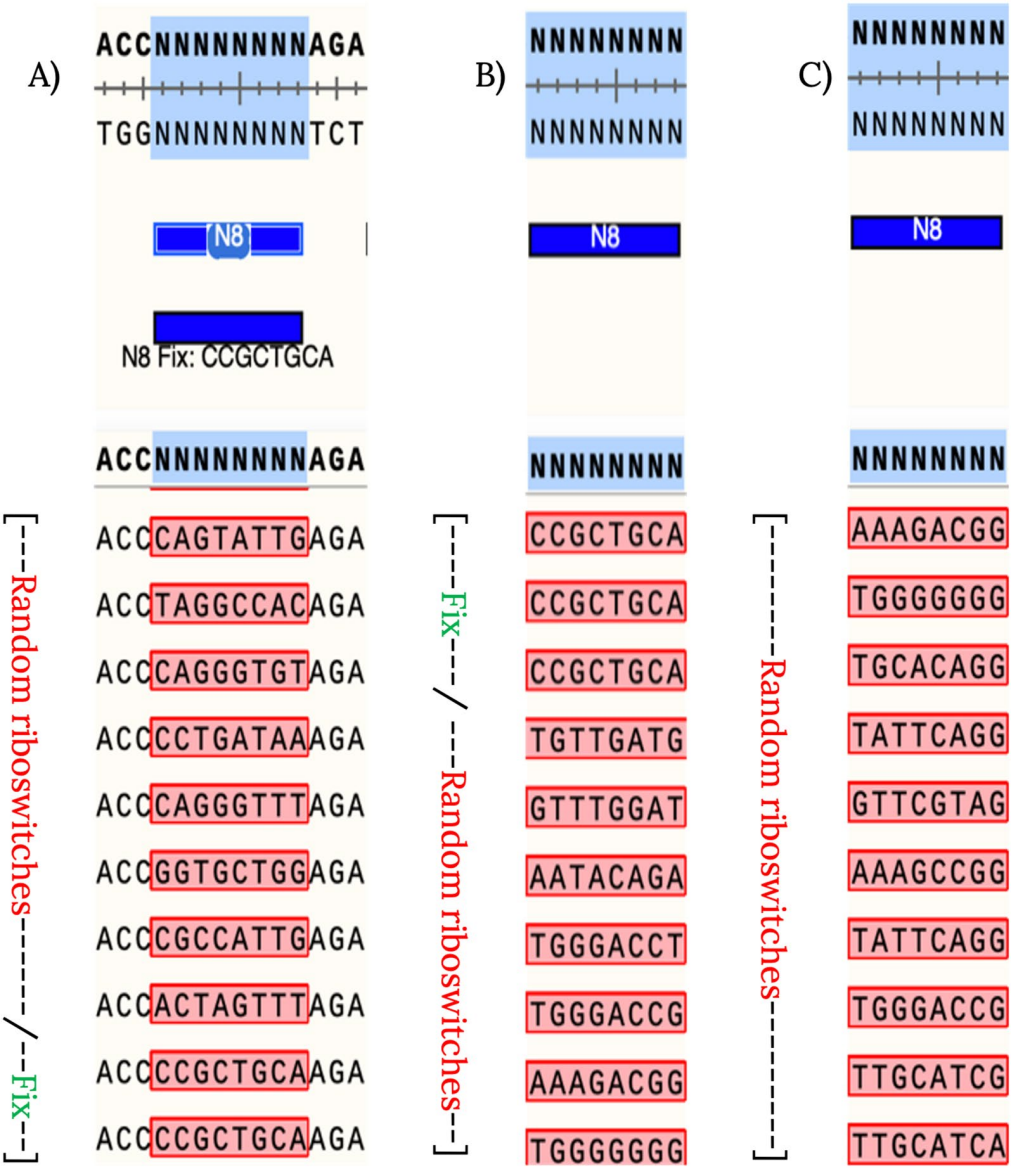
Sequences obtained from initial plasmid and phage libraries*.* Collection of riboswitch sequences obtained during the development of the different libraries. The sequences were obtained through Sanger sequencing of PCR products derived from single colonies carrying plasmids PAJ341 (positive control riboswitch) and PAJ342 (random library of switches) and recombinant phage plaques. The riboswitch sequence used as a positive control (marked as “Fix” in the figure) served as a reference during the initial setup of the procedure. The sequences obtained from the libraries demonstrate the diversity of riboswitch variants generated, laying the foundation for subsequent selection and evolution steps. (**A**) Sequences obtained from single colonies carrying either control or library plasmids. These are only a set of all sequenced colonies, meant to showcase the variation within the plasmid library. (**B**, **C**) Sequences obtained from recombinant phages produced after infecting either a control or a library strain.

To examine the initial diversity of the riboswitch libraries, we conducted sequencing analyses on samples taken from the various plasmid constructs and the phages post the homologous recombination process. Sequencing outcomes disclosed the existence of unique versions of the riboswitches in both the plasmid libraries (PAJ341 and PAJ342) and the phage library (Fig. 4). Significantly, no duplicate sequences were discerned in either the plasmid or the phage populations containing the randomized libraries. This attests that our method successfully yielded diverse and non-repetitive riboswitch variants, thereby establishing a robust groundwork for the subsequent selection and evolution process. Figure 4 offers a visual illustration of the sequence diversity observed in the plasmid and phage libraries.

### Dual selection cycles show enrichment with an engineered T7-phage library of randomized riboswitches

After carrying out 16 cycles of positive and negative selections, we selected and analyzed a total of 24 phage libraries from different stages in the process to assess the sequence variation and evolution of the riboswitch population. During this analysis, we studied smaller library samples comprising 10^5^ reads across the 16 selection cycles. This sample size was deemed appropriate as it encompassed the maximal diversity of the riboswitch library, containing 65,536 variants. The selection process entailed using 1.5 mM theophylline for the phage populations before the 10th generation, while 1 mM theophylline was employed for each generation thereafter to impose a more rigorous selection.

**Figure 5:**
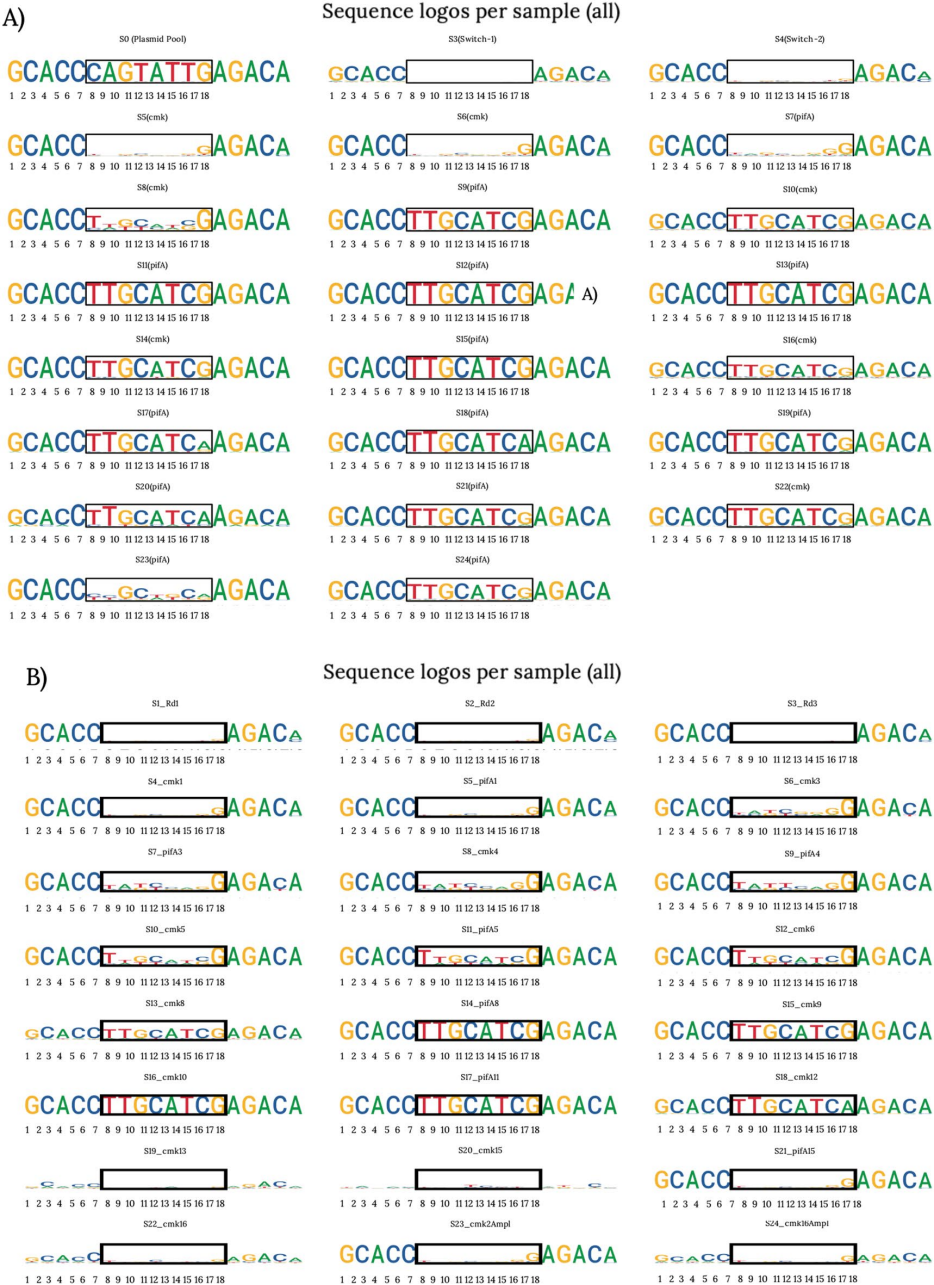
Evolution of predominant riboswitch sequences throughout dual selection cycles. Diagram representing the sequence variation for each tested library, arranged from libraries early into the process (top left) to the most recently produced library (bottom right). Each set corresponds to the predominant sequence observed in the phage population after each selection cycle. The black square indicates the region of the riboswitch sequence that was randomized. The size of each nucleotide in the diagram represents its relative abundance in the sample population. The nomenclature “S+number” indicates the number of selections since the beginning of the project. (**A**) Representation of the first set of libraries that were assessed. Initially, no specific sequence dominance was observed in the early selection cycles (Switch-1 and 2). However, over time, a progressive increase in the dominance of a particular sequence was observed, indicating its successful selection above others. (**B**) Represents a second set of libraries, focusing on the early generations to fill in the gaps observed in (**A**) and evaluate additional phage generations. Note that the last set of logos may have been affected by human error during preparation, which could explain the observed sequence variations. Further details about the process can be found in the “Materials and methods” section, and additional information regarding these experiments can be found in Table S1.

Figure 5A and B illustrate the outcome of the analysis, displaying diagrams of the 8 randomized nucleotides, encased by 5 fixed nucleotides on either side. The size assigned to each nucleotide in the diagram directly reflects the number of times it is present in that position amongst all reads. This thorough analysis unfolded across multiple libraries, as enumerated in Table S1, and spanned diverse phases of the selection trajectory. Each step carries the label “cmk” or “pifA”, identifying its association with either positive or negative selection. In the wake of the selection and evolutionary process, these diagrams afford valuable insights, underscoring the dynamic shifts in nucleotide preferences and mutations within the riboswitch sequences.

Our next-generation sequencing (NGS) data analysis for various libraries yielded intriguing results. As demonstrated in Fig. 5A and B, the initial set of sequences analyzed reveals no overt dominance of any sequence. However, a dwindling variation within each phage population becomes apparent with the advancement of selection steps, suggesting a convergent evolution towards a prevailing sequence. This pattern aligns well with past studies^9,28^ that report a favored nucleotide, such as G, in position 8.

In a subsequent round of NGS scrutiny, most of the sequences echo this pattern of diminishing variation across generations alongside the rise of a dominant sequence. Yet, a subset of libraries (S23 in A and S19, S20, S21, S22, and S24 in B) display a heightened variation. The underpinnings of this amplified variation within these specific libraries currently remain elusive, warranting deeper exploration.

### Estimation of riboswitch activity from phage phenotype shows riboswitch adaptation during evolution

**Figure 6:**
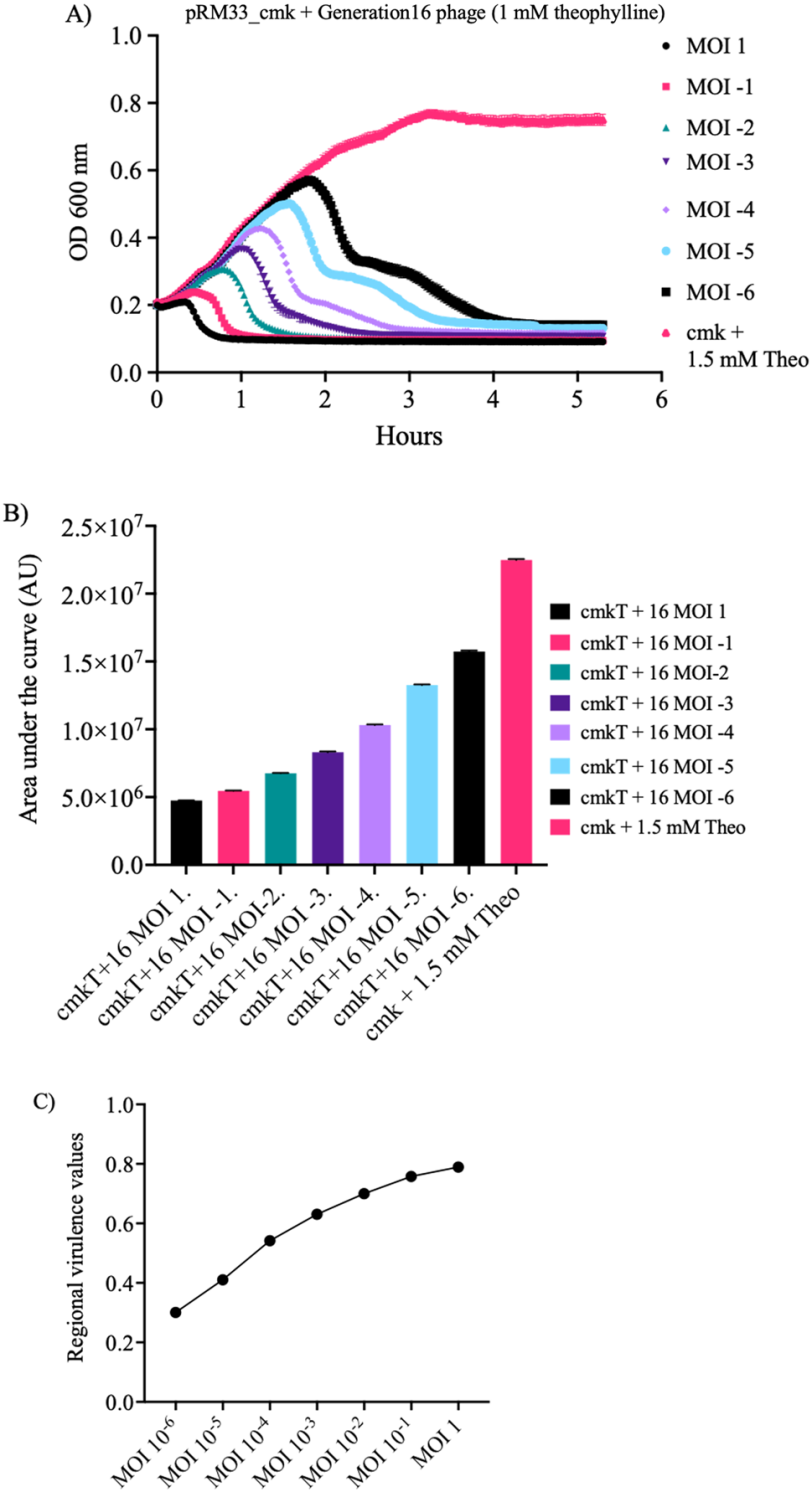
Standard set of data used to obtain the virulence index for a phage population infecting a specific strain. (**A**) Killing curves of positive selection strain cultures with varying multiplicities of infection (MOIs). Each individual curve represents a different cell-to-phage ratio, ranging from 1:1 (MOI 1) to 1^6^:1 (MOI -6), along with the growth curve of the cell strain without phages (which corresponds to MOI = 0). The start of stationary phase is marked as the last timepoint included to calculate the area under each specific curve. (**B**) Calculation of the area under each curve from panel A, represented as Arbitrary Units (AU) on the Y-axis. Each value is what’s known as local virulence, for that phage at that concentration. The cultures labelled as “cmkT” indicate the presence of theophylline in the media. (**C**) Representation of the values obtained from panel (**B**) using Eq. (1), as described in the “Materials and Methods” section. The value for the area under this curve corresponds to the virulence index for each phage in the respective strain. A more in-depth explanation of the calculations is found in the “Materials and methods” section.

We executed a series of virulence index (VI) assays^32^ to substantiate our selection results, as outlined in the “Materials and methods” section. We tested a variety of phage populations procured throughout the process, such as the positive control (a recombinant phage bearing a known, functional riboswitch), phages from generations 2, 5, and 16, and a wild type T7 phage population lacking a riboswitch (negative control). Each population underwent testing under theophylline's presence and absence, employing positive and negative selection strains. Our assays examined OD600 absorbance curves and regional virulence values, enabling us to calculate the virulence index. Figure 6 showcases a sample set of results derived from infecting the positive selection strain with phages subjected to 16 selection cycles.

**Figure 7:**
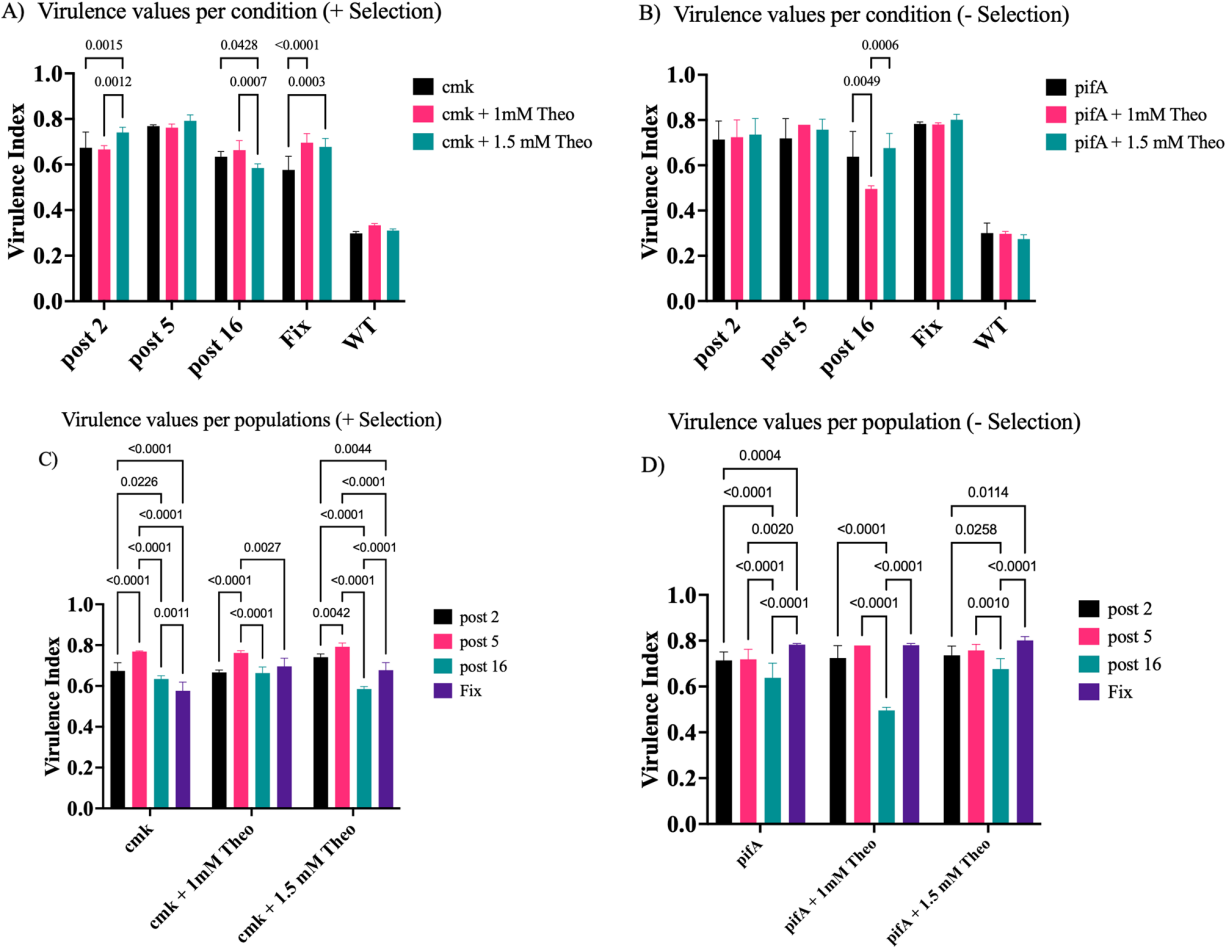
Virulence comparisons between phage populations. Results of the virulence index calculations for different phage populations (Wild Type/Negative control, Control riboswitch/Positive control, and after 2, 5, or 16 cycles of selection) when infecting the positive and negative selection strains in the presence and absence of different concentrations of theophylline. The virulence index values range between 0 and 1, with higher values indicating higher virulence of the phage population. In (**A**) and (**B**) samples are grouped by the phage population used for infection, and in (**C**) and (**D**), by the infected strain. (**A**) The virulence indexes of the five phage populations are shown after infecting the positive selection strain in the absence and presence of theophylline. In the presence of theophylline, the riboswitch changes to an “ON” conformation, leading to higher virulence. (**B**) Virulence index for the different phage populations when infecting the negative selection strain in the absence and presence of theophylline. In this case, virulence is expected to be higher in the absence of theophylline as the riboswitch remains in the “OFF” state. When theophylline is added and the switch turns “ON,” the expression of pifA blocks efficient T7 replication, reducing virulence. (**C**) Differences in virulence between phage populations after infecting the positive strain under different conditions. (**D**) Differences in virulence between phage populations after infecting the negative strain under different conditions. WT was not included for image clarity. The corresponding statistics for the significance of the pair comparisons can be found in Table S3. The significance was determined using unpaired t-tests, and the error bars in panel I were obtained using error propagation.

We evaluated the activation efficiency of riboswitches across diverse phage populations, including wild-type T7, the Positive Control Riboswitch, and samples from cycles 2, 5, and 16 (Fig. 7). We used virulence index values from varying combinations of phages, strains, and theophylline concentrations to ascertain this efficiency. Our results presented unique patterns across these populations. While the wild-type T7 showed no change in its virulence index, the Positive Control Riboswitch generally indicated a higher fold-change in activation. Test samples from cycles 2, 5, and 16 revealed virulence variations. Particularly, in the negative selection step, phage populations from cycles 2 and 5 (selected at 1.5 mM) displayed higher activation rates than the Positive Control. The population from cycle 16 (selected at 1 mM) showed lower efficiency. However, when the riboswitch underwent conformational changes, cycle 16 at 1 mM manifested the most significant activation rate, showing a 28% increase. These observations underscore the variable activation efficiency of riboswitches in different phage populations and underscore the significant role of selection conditions in their performance.

Figure S1^33^ provides a comparative view of the potential impact of changes in riboswitch sequences on virulence through their predicted structures. It not only features random sequences from the initial selection stages and the Positive Control riboswitch sequence, but also the dominant sequences emerging in the selection’s later stages. This comparison serves to underscore the structural variances among the riboswitch variants obtained throughout the selection process and their possible influence on virulence.

## Discussion

Our novel genetic system, T7AE, successfully facilitated riboswitch selection through T7 phages and a dual-selection process in live cells, leading to the selection of a theophylline riboswitch. The sequencing of the resulting phage populations offered a timeline of evolution, pinpointing when specific nucleotides solidified their positions and when a certain sequence rose to dominance in the population (Fig. 5). From our findings and data in Table S1, it is evident that phages surviving the initial selection exhibited a preference for G as the 8th nucleotide in the sequence. By the sixth cycle, the TTGCATCG sequence (or its slightly improved version, TTGCATCA) emerged as the dominant variant, maintaining prevalence through the following generations. This dominant sequence bears notable similarities to the control sequence (CCGCTGCA) from earlier studies^26^, with six of the eight purines and pyrimidines occupying identical positions.

We utilized virulence index calculations to evaluate each phage population's infection efficiency, providing a robust comparative measure among diverse phage populations and a more thorough assessment than growth curves alone^32^. Figure 6 showcases the virulence index values of various phage populations, including two controls: Wild Type T7 and the Control riboswitch^26^ depicted in Fig. 3. We utilized Wild Type T7 to assess switch activation and its virulence impact, while the Control riboswitch served as a functional standard. Predictably, the Wild Type T7 virulence index hovered around 30% (Fig. 7A and B), given the absence of an activatable switch. The positive control phages, used to establish the procedure, exhibited a virulence increase of 20% and 17% in the positive selection, while no difference surfaced in the negative selection.

Despite the observations in Fig. 7 panels A and B, our statistical analysis found that differences between the riboswitch’s ON and OFF states weren't always statistically significant. In the positive selection, the positive control consistently exhibited significant differences between the ON and OFF states, but only at one of the theophylline concentrations. The negative control, in alignment with expectations, showed no significant differences in any situation. Generation 5, among the various phage populations examined, presented no significant differences between the ON and OFF states. However, generations 2 and 16 showed significant differences in virulence values between activation and inactivation.

Only phages from generation 16 were able to show significant differences between different conditions, something not even the positive control phage was able to do. These results demonstrate that random riboswitch populations have undergone selection and adaptations over successive generations, leading to variations in activation efficiencies and virulence, which in some cases can surpass the capabilities of the control switch. To further prove this point, Fig. 7C and D show the significant differences between different phages infecting the same type of culture. The fact that each phage population has a different virulence index for the same strain shows that changes have indeed taken place between generations, which can affect the efficiency of the switch between generations.

Our findings in the negative selection can be attributed to the incomplete understanding of the pifA molecular cascade^34,35^and the potential for system leakage. Given the current limited knowledge of the pifA protein mechanism and the phage exclusion process^34–36^, some effects may go unnoticed during the selection process. This could result in more significant variations between results, especially in the negative selection.

Our examination of the actual test populations and their virulence index (VI) values revealed intriguing patterns when compared to the phage carrying the known control riboswitch sequence. Two of three test populations exceeded the VI values of the control riboswitch sequence. However, as Fig. 7 demonstrates, this increased infectivity didn't correlate with improved riboswitch sequences. Population 5, despite presenting the highest virulence values, consistently showed similar VI values, particularly in the positive selection. This pattern could stem from the prevalence of a sequence perpetually in the "ON" state, sufficiently abundant to outperform other sequences during growth assays. No sequence in generation 5 achieved complete fixation within the population as the NGS analysis (Fig. 5, S8 in A and S10 in B) reveals, hence the improvements in virulence indexes with additional selection steps. Our T7AE genetic system has proven its potential in selecting theophylline riboswitches. The combination of sequencing analysis of phage populations and VI calculations delivers rich insights into the phage population evolution timeline and infection efficiency. Nonetheless, statistical analyses show not all differences between the riboswitch's ON and OFF states are statistically significant, implying the need for deeper exploration of the pifA molecular cascade and possible system leakage. The observed consistency in higher VI values between all populations underscores the selection process's complexity and the need for comprehensive characterization of riboswitch populations to identify the most effective sequences.

Our dual-selection T7AE system, specifically designed to enrich functional riboswitches, effectively identifies efficient riboswitch sequences from a random pool, as evidenced by our virulence index and next-generation sequencing (NGS) data analysis (Figs. 5 and 7). It is important to note, however, that phage virulence does not invariably correlate with riboswitch efficiency. Nonetheless, the riboswitches identified through our system demonstrated improved characteristics over the control riboswitch validated in prior research^26^, as illustrated in Fig. 7. Our study specifically targets mutations at the riboswitch locus, significantly minimizing mutations in other regions, which may only occur during the construction of the phage library. While future studies involving adaptive evolution might introduce mutations outside the riboswitch locus due to the error-prone nature of our T7 DNA polymerase, our dual-selection framework strongly favours the enrichment of functional riboswitches. The likelihood of a phage acquiring random mutations that enhance virulence in both selection environments is remarkably low. Such a scenario would necessitate the development of a novel molecular binder to theophylline through a series of unlikely adaptive mutations. For riboswitches evolved over extended periods, direct measurement of activity using reporter gene assays will provide a final validation.

Our technology could potentially evolve to incorporate the PACEmid approach, using satellite T7 phages, inspired by the T7 phage’s proven efficacy in evolution studies^37^, where only the evolving DNA needs the packaging signal. Our T7AE riboswitch selection approach allows us to discover riboswitches with unique ligand specificities. Our dual selection process establishes a versatile methodology for the phage-assisted accelerated evolution of gene switches. In short, our T7AE system represents a novel approach for riboswitch evolution, with potential to advance riboswitch and biosensor development. The integration of T7 phages and the use of virulence index and NGS data contribute valuable insights into the selection process and resulting riboswitch populations. With further developments and exploration, our T7AE strategy holds significant promise for the production of functional riboswitches and the acceleration of gene switch evolution.

## Materials and methods

### LB media

All bacterial strains were grown in LB media for the different experiments, with any alternative media used only for specific experiments, and described accordingly. The formulation for 1L includes 10 g Peptone 140, 5 g yeast extract, and 5 g sodium chloride, diluted in ddH2O.

### Saline-Magnesium buffer

Solution used to store phage samples, avoiding the risk of contamination by microorganisms^38^. For 1L, 50 mM Tris–HCl 7.5, 100 mM NaCl, 100 mM MgSO_4_ diluted in ddH20.

### Cell strains

Cells from the Keio Collection^39^, were used to transform the confirmed plasmids and to use the resulting cells for the positive and negative selections. Strain specifications are described in the Supplementary Materials.

### Plasmids

Plasmid specifications and assembly details are described in the Supplementary Materials.

### Primers and gBlocks

All gene sequence constructs produced by Integrated DNA Technologies and can be found in Table S2. Melting temperatures (T_m_) of the primers were calculated using the NEB calculator. PCR reactions were carried out using Phusion Master Mix from NEB in a final volume of 25 µL for diagnostic PCRs, and 50 µL for purification PCRs.

### Digestion enzymes

All used enzymes were part of the FastDigest line from Thermo Scientific. All digestions were done at 37 ºC, using 200 ng of DNA template, 1 µL of FastDigest enzymes, and 10× buffer solutions from ThermoFisher in a final volume of 20 µL.

### Polymerase chain reaction

All PCR reactions used for plasmid assembly were done using the 2× Phusion mix from NEB, in a 50 µL volume, according to manufacturer specifications. In cases where the secondary structure of the sequences interfered with the amplification, different percentages of DMSO were used (3, 6%) to help the correct amplification. Diagnostic PCRs from colonies or phage samples were done using a 2× Taq or GreenTaq Mix (Thermo Scientific) under manufacturer’s specifications.

### Goldengate assembly

Based on Engler, Kandzia and Marillonnet^40^. The desired plasmids were amplified via PCR. The primers used contain an overhang not present in the original template, which introduces a BsaI/Eco31I cutting site. Compatible ends were included into the tails of primers used to amplify the desired fragment introduced in the backbone. Using 100 pmol of each DNA template, 1 µL of BsaI (New England Biolabs/NEB), 1 µL of T4 DNA (Fermentas), 2 µL of 10× ligase buffer and H2O up to 20 µL, each reaction tube underwent the following series of cycles: 2 min at 37 °C followed by 5 min at 16 °C, 35 cycles, 5 min at 50 °C and finally 5 min at 80** °C**.

### Agarose electrophoresis

Amplified samples were run in 1% agarose gels^41^, to confirm their size and approximate quantity, at 95 V for 30 min for most experiments. Smaller samples were run in 2% agarose gels.

### Purifications

All PCR reactions were purified using the Thermo GeneJET PCR purification kit. In cases where several bands appeared when running the sample in a gel, the GeneJET Gel extraction kit was used.

### Transformations

Plasmids were transformed into both electrically and chemically competent cells. For transformations in electrically competent bacteria, a minimum of 100 ng of DNA were added to 50 µL of competent cells and left on ice for 15 min, along with an electroporator cuvette. The volume was then transferred to said cuvette, the cells electroporated and then grown in LB media for 1 h before being plated. For chemically competent cells, a minimum of 100 ng of DNA was added to the 50 µL of cells, which were left on ice for 30 min, then heat shocked at 42 ºC for 90 s, and returned to ice for 10 min before recovery in LB media for an hour at 37 ºC. Cells were then plated in an LB agar plate with their corresponding antibiotic resistances.

### Growth

To obtain cell cultures for the various assays, colonies picked from grown plates were grown overnight in 5 mL of LB with the appropriate antibiotic, using Falcon 15 mL round bottom tubes. In the cases of positive selection strains, to prepare them for the assays, 300 µL of 25 mM Theophylline were added to the 5 mL of LB. Overnight cultures were refreshed the next day and used when an OD between 0.2 and 0.3 was reached. The remaining overnight culture was used to create glycerol stocks.

### Phage killing and homologous recombination

To obtain phage populations or test their killing efficiencies, phages were added to cultures within the growth phase (OD 0.2–0.3), making sure the phages would be able to properly replicate. In cases where the objective was to produce homologous recombination, a rate of 1 phage per 10^5^ cells was used. These tests required phages to infect ∆*trxA* cells containing gp5/T7 DNA polymerase. This ensured that only recombinant phages would be able to replicate, as trxA is an essential protein for the process^24^. All lysates were then centrifuged for 10 min at 3000 rpm and filtered with 2 µm filters, obtaining clean phage samples.

### Selection procedure

8 different strains were tested for each of the selection steps, using the control riboswitch to find the most efficient strain to be used for evolution. This was done by infecting each of the strains of interest with the recombined T7 phages in the following ways:

- Growth assays: In a 96-well plate, each well is filled with 180 µL of cells at an OD of 0.2–0.3, and 20 µL of the tested phages in a decreasing order along the length of the plate. In the case of cultures with theophylline, two ways were tested: 180 µL of a culture already containing theophylline, or 163.5 µL of culture plus 12.5 µL of 25 mM theophylline, to which the phages were added. As a way to keep all records the same, and have a consistent concentration in the cultures, the first method was preferred. The plate reader (model, make) took OD measurements every 2 min for a duration of 5 h. To compare the differences between strains and select the best candidates for the selection and evolution procedure, the growth curves for each cell strain were plotted using Microsoft Excel and Graphpad Prism 9.
- Plaque assays: These assays were developed to check the replication efficiency of the phages^42^. By infecting 300 µL of cells at an OD of 0.2–0.3 with 100 µL of phage at different dilutions, mixing it with 3 mL of soft LB agar (LBA 50% in LB), and then plating it, clear spots or “plaques” can be observed in the bacterial lawn of the plate. A higher number of plaques indicates a higher number of phages, indicating which strains were better for the replication of the phages. These assays were made both in the presence and absence of 1.5 mM Theophylline, to assess the “leakiness” of the system and the activation of the riboswitch.
- One-Step assay: These assays (Fig. S2) follow the same procedure as a plaque assay, but phage samples were obtained from infecting a culture^43^. Samples were taken every 4 min over a period of 40 min, showing the rise in the number of phages as the infection progresses. The assays were also made in the presence or absence of 1.5 mM theophylline to determine the differences due not only to the two types of sample treatment, but to the addition of the activating molecule to the media, thus affecting the phages’ replication process. These phage samples were then used for plaque assays as described previously.

### Next-generation sequence analysis

This strategy was designed following the 16S amplicon sequencing protocol from Illumina, using its Nextera XT DNA Library Kit (Illumina #FC-131-001) to prepare the samples for sequencing in an Illumina MiSeq (Fig. S2). A first PCR was carried out to amplify a 175 bp fragment from the phage genome containing the closest elements present upstream and downstream from the region containing the 8 randomized nucleotides of the riboswitch (Fig. S3A). The primers used in this PCR also incorporated sequences necessary to carry out the indexing PCR for the sequencing (Fig. S3B). Combinations of primers for the Index PCR are shown in Table S2. The final amplicon is shown in Fig. 3C.

### Riboswitch sequence analysis

Due to the lack of available computing power, the initial set of analyses were done in a fraction of the total number of sequences, approximately 10^5^ per sample.

Sequence quality control was performed using the FastQC software^44^. After trimming, all reads corresponding to each sample were aligned to the initial “parent” sequence (as designed and cloned into the initial vector prior to selection) using a recursive Needleman-Wunsch pairwise alignment algorithm^45^. Once aligned, all reads were trimmed from both ends until only the 8 nt-long riboswitch sequence plus 5 bases on either side remained. Trimming was performed using base R, by finding the 8 nucleotide long “NNNNNNNN” motif in the parent sequence and extracting this position (± 5 bp) for all reads in each sequence. Once extracted, nucleotide proportions were calculated by stacking each position and counting the number of each nucleotide that was informative (not ambiguous), which is considered a Bit. The program does not only count which percentage of each nucleotide is present in every position, as there are many reads showing an ambiguous read or just a gap in these positions. To account for this, it creates a list and only counts the number of times every base is clear, represented as an informative Bit, according to information theory. The entire process of nucleotide counting and plotting was performed using the R package *ggseqlogo*^46^.

### Population evolution assessment via virulence index calculation

The selected procedure shown previously^32,47^, was used, by which a value known as the virulence index was ascribed to different phages to compare them to one another. The virulence index was obtained via a two-step process:

1. Calculating the local virulence ($\upsilon 1$) via the following formula:
  1 $$ \upsilon {1} = {1} - \frac{Ai }{{A0}} $$
  where A_0_ represents the area under the growth curve of a cell culture without phages until reaching stationary phase, and A_i_ the area under the curve of a culture at a certain MOI until the same timepoint. $\upsilon 1$ is measured in a range of 0 to 1, with 0 being no virulence whatsoever, and 1 being the maximum theoretical virulence, instantaneous death of the infected cells.
2. Once the local virulences over a range of MOIs were assessed, they were represented on a curve. The area under that curve (A_p_) was calculated following the formula
  2 $$ {\text{A}}_{{\text{p}}} = \mathop \smallint \limits_{i}^{0} \upsilon 1{\text{d}}(\log MOI) $$
  with *i* being the base10 log of the lowest MOI, which in this case was − 6.
3. That value was then divided by the theoretical maximum value (A_max_) for the same curve, giving the virulence value for a specific phage on a specific strain. In this case, this maximum value corresponded to 6.
4. These values were readily comparable with each other, and were taken both as they were, and to calculate the “activation efficiency” for the riboswitches inside the phages, indicating the fold increase between states of the switch in those cases.

The virulence index measurements were made for 5 different phage populations: 3 at different stages of selection, 1 for the original riboswitch-carrying phage, and one for WT T7. Each of these phages was used to infect the positive and negative selection strains under three conditions: 0 mM, 1 mM and 1.5 mM Theophylline; to assess their virulence under each condition. All calculations were made using Microsoft Excel 2019 and Graphpad Prism 9. Statistical significance between the different virulence indexes was done via unpaired t-tests.

### Supplementary Information

Supplementary Information 1.

Supplementary Information 2.

## Data Availability

All data generated or analysed during this study are included in this published article [and its supplementary information files].

## Abbreviations

*cmk*: CMP kinase
trxA: Thioredoxin A
MFE: Minimum free energy
MOI: Multiplicity of infection
PACE: Phage Assisted Continuous Evolution
SELEX: Systematic Evolution of Ligands by Exponential enrichment
