# Supplementary material for: T7 phage-assisted evolution of riboswitches using error-prone replication and dual selection
Source: Sci Rep. 2024 Jan 29;14:2377. doi: 10.1038/s41598-024-52049-9 (PMC10824729; doi:10.1038/s41598-024-52049-9)
Supplement: Supplementary file 1 — Supplementary Information 1. [file 41598_2024_52049_MOESM1_ESM.docx]

**Supplementary Materials**

**T7 phage-assisted evolution of riboswitches using error-prone replication and dual selection**

**Authors**

Eduardo Goicoechea Serrano^1,2^, Alfonso Jaramillo^1,3*^ and Carlos Blázquez-Bondia^1†^

**Affiliations**

^1^ Warwick Integrative Synthetic Biology Centre and School of Life Sciences, University of Warwick, Coventry CV4 7AL, UK.

^2^ London BioFoundry, Imperial College Translation & Innovation Hub, White City Campus, 84 Wood Lane, London W12 0BZ, United Kingdom

^3^ *De novo* Synthetic Biology Lab, i2sysbio, CSIC-University of Valencia. Parc Científic Universitat de València. Calle Catedrático Agustín Escardino, 9. 46980 Paterna. Spain.

† Deceased

* To whom correspondence should be addressed:

Prof. Alfonso Jaramillo. *De novo* Synthetic Biology Lab. I2SysBio, CSIC-University of Valencia. Parc Científic Universitat de València. Calle Catedrático Agustín Escardino, 9. 46980 Paterna. Spain. <https://orcid.org/0000-0002-6313-9689>,

[Alfonso.Jaramillo@synth-bio.org](mailto:Alfonso.Jaramillo@synth-bio.org).

**Supplementary Materials and Methods**

Strain List: Cells from the Keio Collection (1), namely the parental strain BW25113 (SAJ128), and KO for *trxA* (SAJ130) and both genes *cmk* and *trxA* (SAJ19) (CGSC, <http://cgsc2.biology.yale.edu)>. The Keio Collection cells were used to transform the confirmed plasmids and to use the resulting cells for the positive and negative selections.

Plasmid List:

Below is a list of the different plasmids utilized throughout the project, with their official denomination, their name throughout the project, and the serial number in the lab’s records. Their specific assembly is discussed in the next section.

- Pr100 pLit Chlor/EG_001/PAJ343: 3494 bp, with a pMB1 origin of replication and Chloramphenicol resistance. Genes 4.7 and 5.3 from the T7 phage’s genome; both flanking a T7 promoter followed by the gene for thioredoxin A to incorporate them via homologous recombination.

- pSEVA631/EG_002/PAJ86: 3001 bp, containing the default multiple cloning site flanked by an rrnB T1 terminator and a lambda t0 terminator, a pBBR1 origin of replication, and a Gentamicin resistance. This plasmid was used as the backbone for all selection plasmids.

Parental positive selection plasmid (EG002+): 7135 bp

Parental negative selection plasmid (EG002-): 8677 bp

- HRF plasmid/EG_cI01/PAJ341: 3569 bp, based upon the ∆*cmk* version of Pr100 pLit Chlor. Via the use of GoldenGate assembly, a gene fragment containing a T7 terminator, a T7 promoter, and a fixed version of the theophylline riboswitch with a high-fold activation, taken from (2), as seen in Figure 1B. This plasmid was used as a positive control for the efficiency of the method.

- HRR plasmid/EG_cI02/PAJ342: 3569 bp. Exactly the same elements as EG_c01, but with the riboswitch contains a randomised region of 8 nucleotides after the stem of the riboswitch and before the RBS (Figure 1C). Thus, a plasmid library of riboswitches containing theoretically 65,536 variants. These will be used for experiments once the method has been properly set up for the fixed version of the riboswitch.
- Positive selection variants: Based upon the positive selection plasmid, different combinations of promoter and RBS were tested to select for the ideal one to use in the current experimental design. This meant a combination of promoter and RBS that gave very low production of *cmk* for a riboswitch in an “OFF” state (without theophylline), and a high one for an “ON” state riboswitch (with theophylline). 8 variants were used, 6 of them test strains, and 2 of them used as controls.
  - The positive control/EG_cI03/SAJ782 (6093 bp) contained a T7 promoter and a strong synthetic RBS in control of the *cmk* gene, ensuring its expression and the proper replication of T7 phages, regardless of the presence of theophylline.
  - The negative control/EG_cI10/SAJ789 (6054 bp) contained neither promoter nor RBS in front of *cmk,* so phages would not be able to replicate under any circumstances, as the transcription system could not be initiated in this case
  - The test variants/EG_cI04-09/SAJ783-8 (6155 bp) contained the previously described pRM promoter followed by 1 of 6 different RBSs of different strengths. These RBSs were known as B0030, B0031, B0032, B0033, B0064, and B00Syn (2).
- Negative selection variants: As in the positive selection, 8 variants were tested, 2 of them being controls and 6 of them being tests. All the variations are as previously described, except for the plasmids containing *pifA* instead of *cmk.*
  - Positive control/EG_cI11/SAJ700: 7635 bp.
  - Negative control/EG_cI18/SAJ707: 7600 bp.
  - Test variants/EG_cI12-17/SAJ701-6: 7700 bp.

Plasmid assembly specifications:

All the constructs assembled were based on previously available plasmids present in the laboratory’s stock. The assemblies were made either by enzymatic digestion at 37 ºC and overnight ligation at 20ºC, or using the process known as GoldenGate ligation.

- PAJ343: The Homologous Recombination plasmid was based on a pre-existing plasmid present in the lab, Pr100 pLit Chlor, itself based on pLITMUS 28 (https://www.addgene.org/vector-database/1528/); and containing the genes for CMP/dCMP kinase and thioredoxin A, making it 4226 bp. The plasmid was modified further before assembling the final version, eliminating the *cmk* gene via enzymatic digestion, making it 3490 bp.
- PAJ86: Based upon the European standard of SEVA plasmids (3), allowing for easy to reproduce modularity. This original plasmid underwent further modifications to assemble the positive and negative selection plasmids. Such modifications included the addition of an error-prone version of T7 phage’s DNA polymerase controlled by a T7 promoter in the 5’ direction, and BsaI sites flanking an R0010 promoter and Ribosome Binding Site (From here onwards RBS) controlling RFP in the 3’ direction, followed by B0010 and B0012 terminators; followed by the selection gene (*cmk* or *pifA* for the positive and negative selections, respectively).
- Selection plasmids: All different cargos were introduced into EG002 via Goldengate assembly, using the previously mentioned GoldenGate cutting sites

**Supplementary Figures**


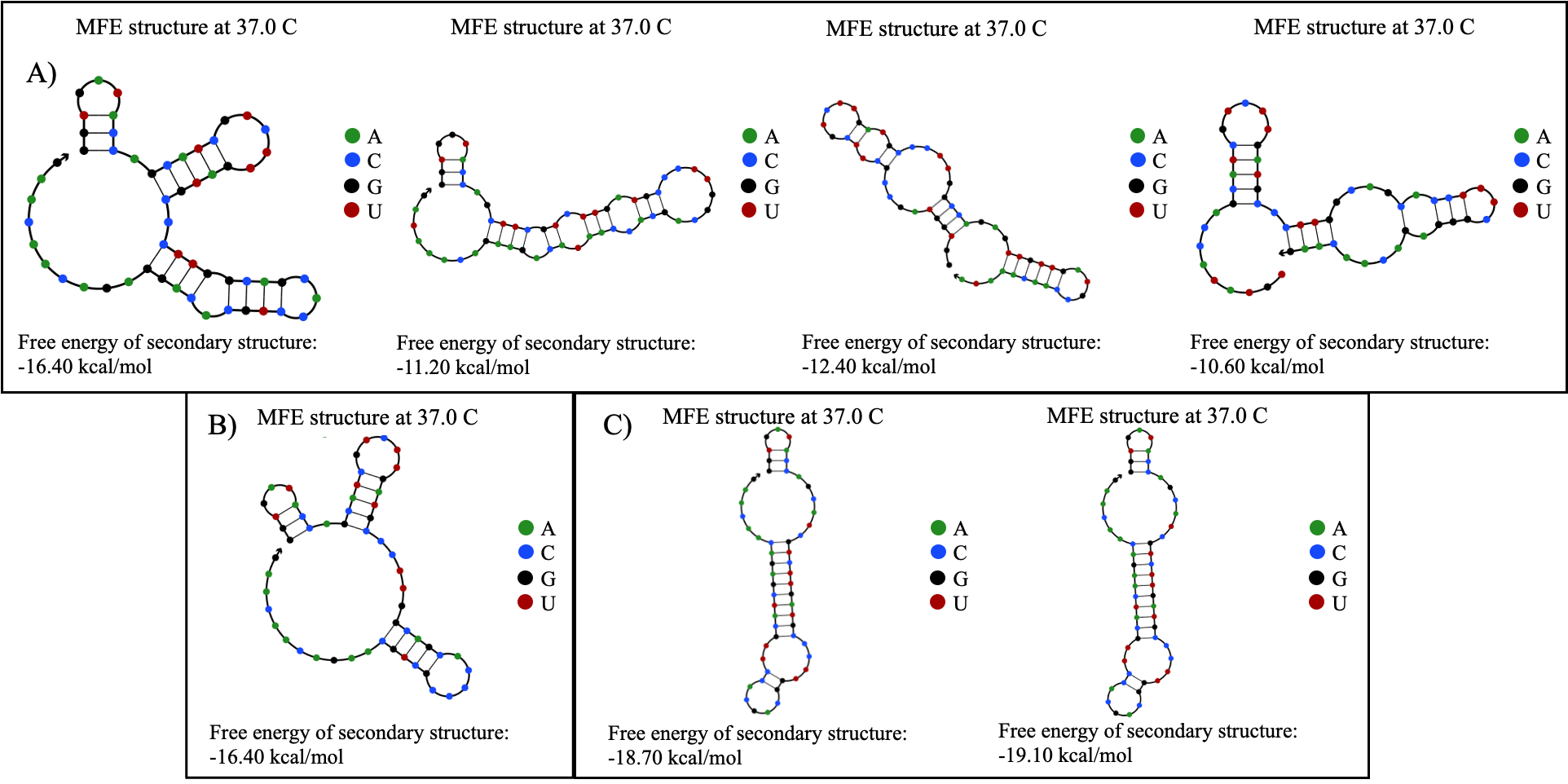


**Figure S1. Structural comparison between riboswitch sequences.** Using NUPACK (4), the secondary structures corresponding to different sequences throughout the selection process were predicted, as well as their MFE (minimum free energy). **A)** Sequences from the random pool of riboswitches from the early stages in the selection. **B)** Riboswitch used as a standard and positive control, with a fixed sequence (CCGCUGCA). **C)** Sequences that were overly represented by the final steps of the selection, as seen in Figure 3B. Left structure: TTGCATCG, Right structure: TTGCATCA. This shows that starting from a random pool of switches, we have been able to obtain sequences which managed to rival and even surpass the stability of the positive control switch.


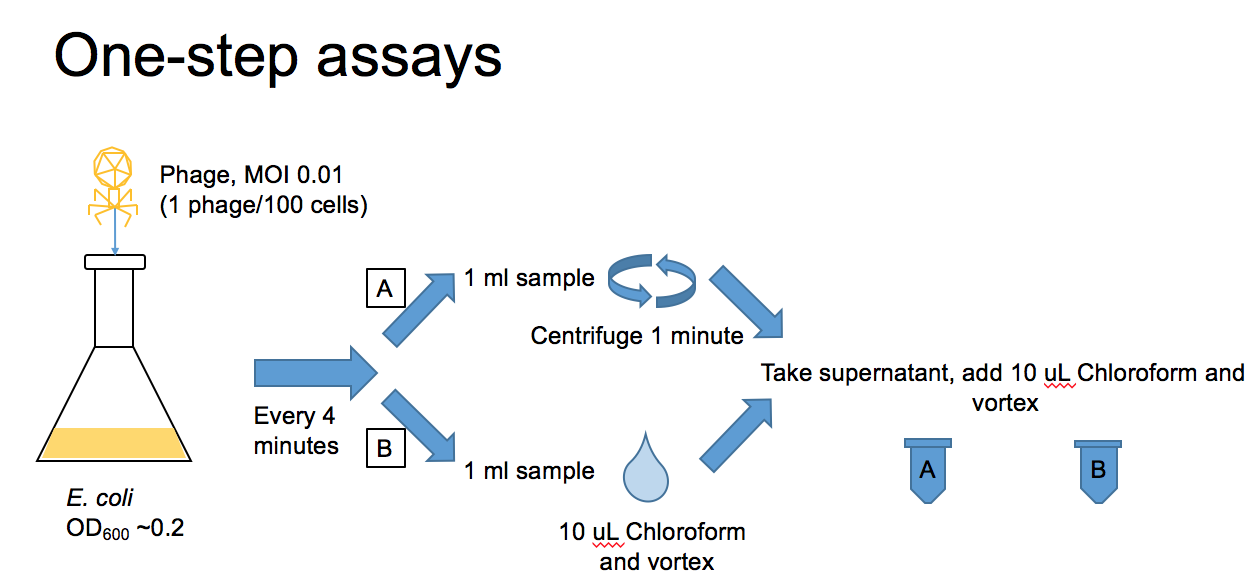


1 ml sample

1 ml sample

Centrifuge 1 min

Phage, MOI 0.01

(1 phage/100 cells)

B

A

Take supernatant, add 10 µL chloroform and vortex

10 µL chloroform and vortex

Every 4 minutes

*Escherichia coli*

OD_600_ ~0.2

**Figure S2. General diagram of a one-step assay**. Once the phages, at a MOI (Multiplicity of Infection) of 10^-2^ (1 phage/100 cells) have been added to the culture, samples are taken and undergo different treatments. “A” are centrifuged to pellet the cells and obtain the phages present in the supernatant, while “B” samples have Chloroform added to them, causing bacteria to burst and release phages that had not yet exited the cells. To eliminate any remaining bacteria present in the final samples, Chloroform is added again, and those phage samples are then used for a plaque assay, where a difference should be observable between different samples and time points.


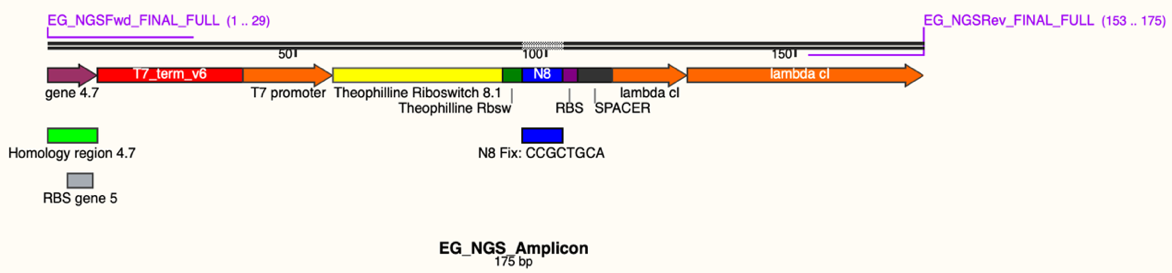


A)


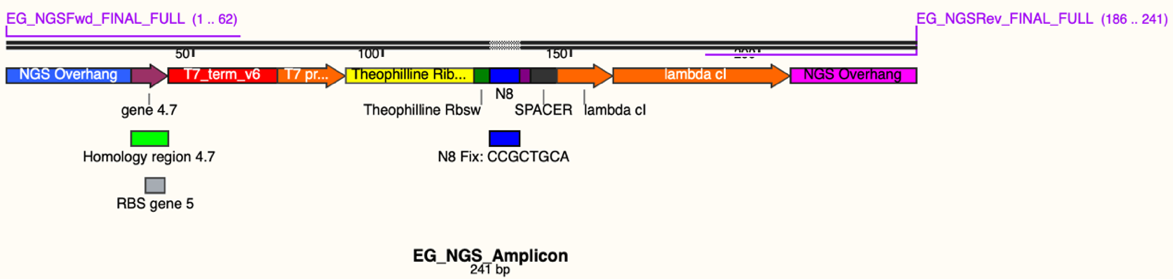


B)


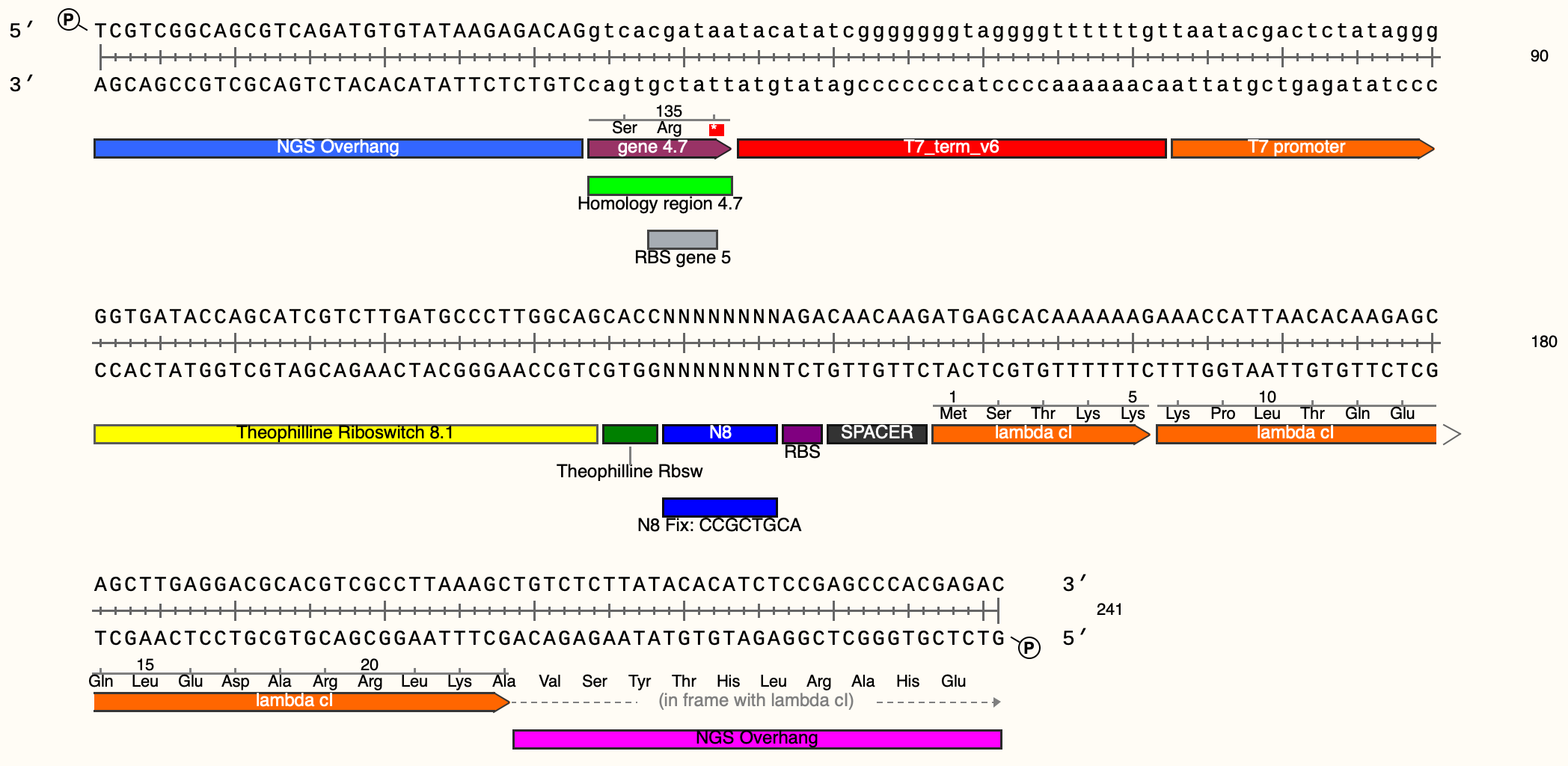


C)

**Figure S3. Amplification steps for NGS preparation.** Selected amplicon to be used for the NGS procedures. A) Original amplicon as it is present in the recombined phages’ genome, 175 base pairs in size, containing fragments upstream and downstream from the random riboswitch sequence. B) Amplicon obtained after the PCR, with the desired primers having added the required Illumina overhangs for the Indexing PCR. C) Detailed sequence of the final amplicon, indicating the original sequences and the overhangs.

*
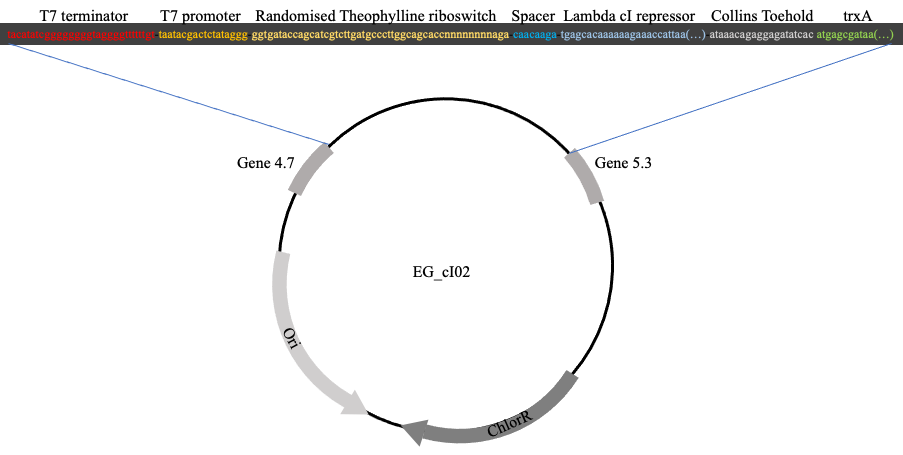
*

**Figure S4. Schematic representation of HR plasmid EG_cI02.** This is the version of the homology plasmid that was used for the creation of the library, carrying the fragment of interest flanked by two T7 genes to act as homology regions.

| **SAMPLES A** | **NUMBER** | **SAMPLES B** | **NUMBER** |
| --- | --- | --- | --- |
| **PLASMID Pool** | S0 | **Rd Switch 1** | S1 |
| **Fix Switch** | S2 | **Rd Switch 2** | S2 |
| **Rd Switch 1** | S3 | **Rd Switch 3** | S3 |
| **Rd Switch 2** | S4 | **cmk1** | S4 |
| **Cmk1** | S5 | **pifA1** | S5 |
| **cmk2** | S6 | **cmk3** | S6 |
| **pifA2** | S7 | **pifA3** | S7 |
| **cmk5** | S8 | **cmk4** | S8 |
| **pifA6** | S9 | **pifA4** | S9 |
| **cmk7** | S10 | **cmk5** | S10 |
| **pifA7** | S11 | **pifA5** | S11 |
| **pifA9** | S12 | **cmk6** | S12 |
| **pifA10** | S13 | **cmk8** | S13 |
| **cmk11** | S14 | **pifA8** | S14 |
| **pifA11** | S15 | **cmk9** | S15 |
| **cmk12** | S16 | **cmk10** | S16 |
| **pifA12** | S17 | **pifA11** | S17 |
| **pifA12B** | S18 | **cmk12** | S18 |
| **pifA13** | S19 | **cmk13** | S19 |
| **pifA13D** | S20 | **cmk15** | S20 |
| **pifA13B_8-11** | S21 | **pifA15** | S21 |
| **cmk14** | S22 | **cmk16** | S22 |
| **pifA14_6-11** | S23 | **cmk2 Amplif.** | S23 |
| **pifA14** | S24 | **cmk16 Amplif.** | S24 |

**Table S1. NGS sample nomenclature.** Correspondence between the generation number of the different analysed libraries and the nomenclature used in the sequence variation diagram. cmk or pifA indicated whether the sample corresponded to a positive or negative step in the selection, and the number indicated the specific generation.

.

| **A)** | **N701** | **N702** | **N703** | **N704** | **N705** | **N706** |
| --- | --- | --- | --- | --- | --- | --- |
| **S502** | PLASMID Pool | Fix Riboswitch | Rd Switch 1 | Rd Switch 2 | Cmk1 | cmk2 |
| **S503** | pifA2 | cmk5 | pifA6 | cmk7 | pifA7 | pifA9 |
| **S504** | pifA10 | cmk11 | pifA11 | cmk12 | pifA12 | pifA12B |
| **S517** | pifA13 | pifA13D | pifA13B_8-11 | cmk14 | pifA14_6-11 | pifA14 |
| **B)** | N701 | N702 | N703 | N704 | N705 | N706 |
| **S502** | Rd1 | Rd2 | Rd3 | Cmk1 | pifA1 | cmk3 |
| **S503** | pifA3 | cmk4 | pifA4 | cmk5 | pifA5 | cmk6 |
| **S504** | cmk8 | pifA8 | cmk9 | cmk10 | pifA11 | cmk12 |
| **S517** | cmk13 | cmk15 | pifA15 | cmk 16 | cmk2 Ampl | cmk16 Ampl |

**Table S2. Primer combinations for each NGS sample.** Matrix indicating which primers correspond to each specific phage library in each of the two repetitions. The 6x4 matrix gives the possibility of separating between 24 samples at once, each of them with their own specific signal, thanks to the specific indexes present in each of the 2 primers used for the amplification. The different denominations for each library are based on the selection step they were obtained from, followed by the number of the generation. If there are multiple libraries corresponding to the same generation, a letter or date is included after the denomination. E.g. Sample cmk11 corresponds to the phage library obtained after the 11^th^ positive selection step in the evolutionary process. A and B correspond to the 1^st^ and 2^nd^ set of sequenced libraries, respectively.


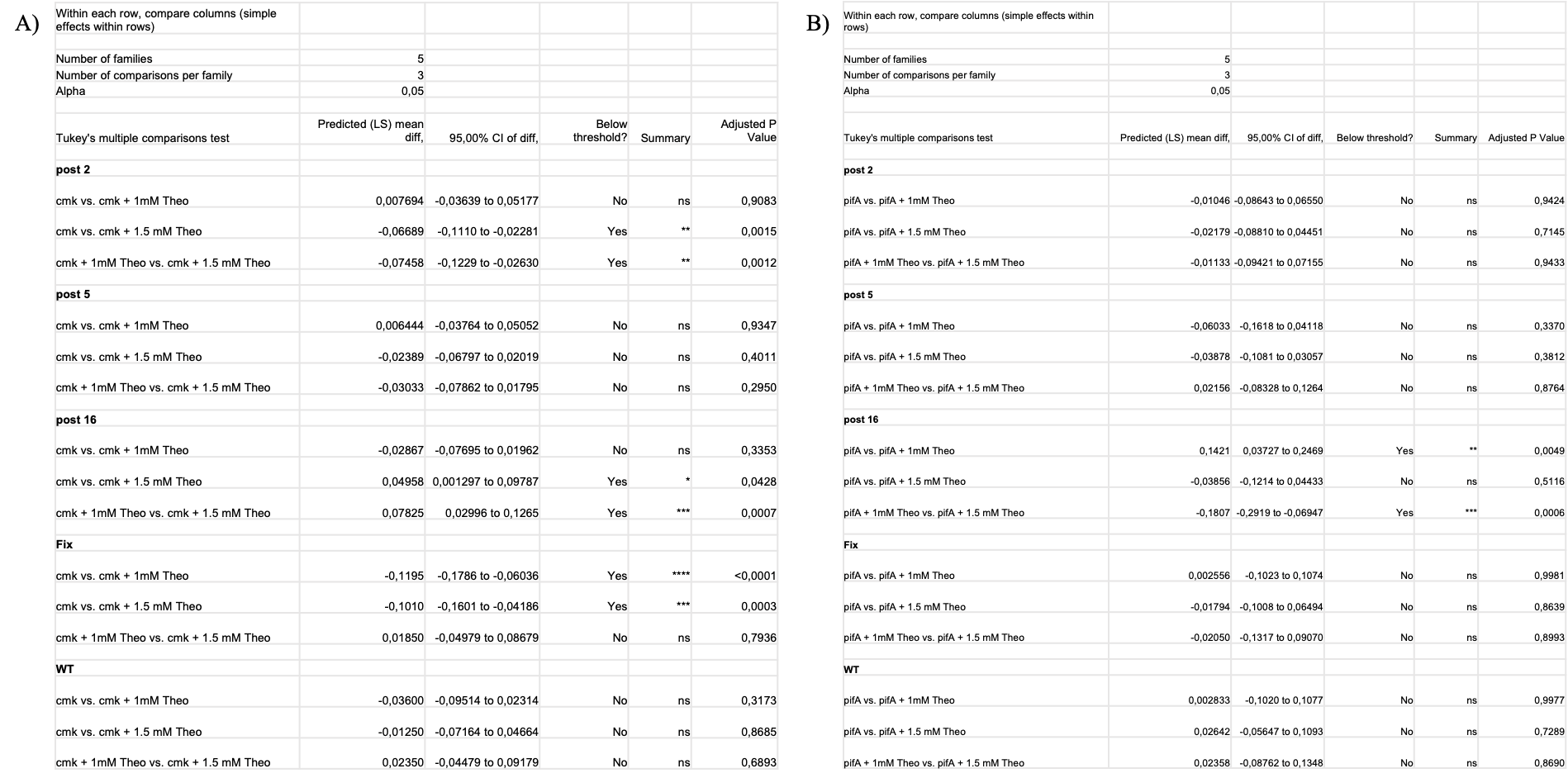

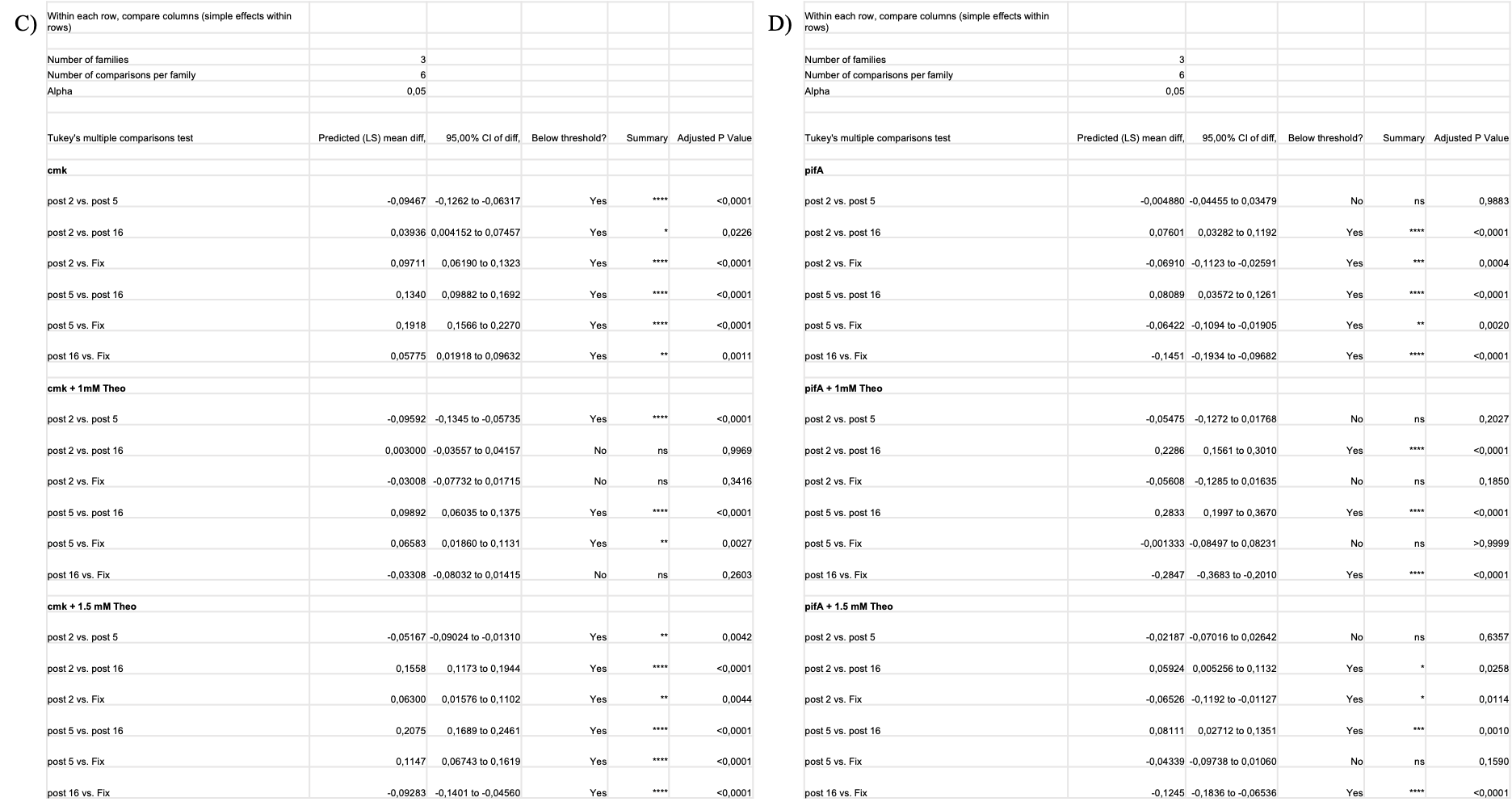


**Table S3. Full statistical data for virulence index significance.** Explanation of the type of statistical analysis and corrections made to determine whether the differences between different populations were significant. **A)** Positive selection, grouped by phage population. Showing the pair comparison between all different populations: cells grown in the absence of theophylline (cmk), cells grown in 1mM theophylline (cmk + 1mM Theo), and cells grown in 1.5 mM theophylline (cmk + 1.5mM Theo). **B)** Negative selection, grouped by phage population. Showing the pair comparison between all different populations: cells grown in the absence of theophylline (pifA), cells grown in 1mM theophylline (pifA + 1mM Theo) and cells grown in 1.5 mM theophylline (pifA + 1.5 mM Theo). C) Positive selection, grouped by growth conditions. Showing the pair comparison between all different populations: cells grown in the absence of theophylline (pifA), cells grown in 1mM theophylline (pifA + 1mM Theo) and cells grown in 1.5 mM theophylline (pifA + 1.5 mM Theo). **D)** Negative selection, grouped by growth condition. Showing the pair comparison between all different populations: cells grown in the absence of theophylline (pifA), cells grown in 1mM theophylline (pifA + 1mM Theo) and cells grown in 1.5 mM theophylline (pifA + 1.5 mM Theo). All calculations were done with Graphpad Prism 9.

1. Baba T, Ara T, Hasegawa M, Takai Y, Okumura Y, Baba M, et al. Construction of Escherichia coli K-12 in-frame, single-gene knockout mutants: The Keio collection. Mol Syst Biol. 2006;2.

2. Weiss R. Cellular computation and communications using engineered genetic regulatory networks. 2001. Available from: http://dspace.mit.edu/handle/1721.1/8228

3. Silva-Rocha R, Martínez-García E, Calles B, Chavarría M, Arce-Rodríguez A, De Las Heras A, et al. The Standard European Vector Architecture (SEVA): A coherent platform for the analysis and deployment of complex prokaryotic phenotypes. Nucleic Acids Res. 2013;41(D1):666–75.

4. Zadeh JN, Steenberg CD, Bois JS, Wolfe BR, Pierce MB, Khan AR, et al. Software News and Updates NUPACK: Analysis and Design of Nucleic Acid Systems. J Comput Chem. 2011;32:170–3.

**Author contributions**

E.G.: Designed research, performed research, analyzed the data, designed figures, wrote the manuscript.

A.J.: Designed research, supervised research, wrote the manuscript.
