# Supplementary material for: T7 phage-assisted evolution of riboswitches using error-prone replication and dual selection
Source: Sci Rep. 2024 Jan 29;14:2377. doi: 10.1038/s41598-024-52049-9 (PMC10824729; doi:10.1038/s41598-024-52049-9)
Supplement: Supplementary file 2 — Supplementary Information 2. [file 41598_2024_52049_MOESM2_ESM.docx]

**Supporting information**

The supporting information includes a list and description of used plasmids and their assembly methods, and a list of relevant primers and their sequences, as well as diagrams of several experimental procedures and nomenclatures.
